# Laboratory evidence on vector competence of the invasive mosquito *Aedes koreicus *[*Hulecoeteomyia koreica*] for *Dirofilaria immitis*

**DOI:** 10.1186/1756-3305-7-S1-O34

**Published:** 2014-04-01

**Authors:** F Montarsi, S Ciocchetta, S Ravagnan, G Simonato, F Mutinelli, S Camuffo, A Frangipane di Regalbono, G Capelli

**Affiliations:** 1 Istituto Zooprofilattico Sperimentale delle Venezie, Legnaro (Padua), Italy; 2 Department of Animal Medicine, Production and Health, University of Padua, Legnaro (PD), Italy

## 

*Aedes *(*Finlaya*) *koreicus *is an exotic invasive mosquito detected for the first time in Italy in 2011. Little information on its vector competence for *Dirofilaria immitis *are available. The area where this species is now established (Veneto Region, north-eastern Italy) is endemic for dirofilariosis. In this study, *Ae*. *koreicus *specimens were experimentally infected with *D. immitis *to evaluate the development of filarial larval stages in different body districts.

*Aedes koreicus *were reared under laboratory standard condition (temperature: 25 ± 1°C; relative humidity: 65 ± 5%; light-dark: 16-8 h). A test group (T) (n = 54 mosquitoes) and a control group (C) (n = 29 mosquitoes) were fed by an artificial feeding system (Hemotek™) using uninfected (in C) and naturally infected (in T) dog blood (3000 microfilariae/ml). Mosquitoes naturally dead and specimens killed at 1, 13, 16, 22 and 28 days post infection (dpi) were dissected; head, thorax and abdomen were examined separately. Five specimens (3 from T and 2 from C) were selected for histology. In addition, molecular confirmation by real time PCR for Dirofilariae were performed. Each larval stage was documented by pictures and videos.

The experiment lasted 28 days. A total of 46 mosquitoes fed in T (85%) and 24 mosquitoes in C (83%) groups. In T, 11 mosquitoes were killed and 32 were recovered dead. The mosquito mortality rate in T was 52% during the first nine days, significantly higher compared to C (8%) (p < 0.01). In total, 31 mosquitoes (67%) were infected. The average of microfilariae, L1 (sausage stage) and L3 was 14.67, 8.56 and 3.15, respectively. Second stage larvae were observed only once (8 specimens on 13 dpi). First stage larvae were first observed on 3 dpi whereas L3 on 8 dpi. The latter were found in salivary glands and proboscis starting on 16 till 28 dpi.

*Aedes koreicus *seems to be a suitable intermediate host for *D. immitis*. Despite the low initial number of microfilariae, the infective L3 stage was observed in all body districts including the proboscis. An high mosquito mortality occurred during the first days, nevertheless one third of them survived and became infective. This results show that *Ae. koreicus *may be involved in the natural cycle of *D. immitis*, increasing the risk of exposure for dogs and humans.

## Funding

This work was sponsored by the Veneto Region and IZSVe.

